# Addressing implementation challenges during guideline development – a case study of Swedish national guidelines for methods of preventing disease

**DOI:** 10.1186/s12913-014-0672-4

**Published:** 2015-01-22

**Authors:** Linda Richter-Sundberg, Therese Kardakis, Lars Weinehall, Rickard Garvare, Monica E Nyström

**Affiliations:** Department of Public Health and Clinical Medicine, Epidemiology and Global Health, Umeå University, SE 90185 Umeå, Sweden; Department of Learning, Informatics, Management and Ethics, Medical Management Centre, Karolinska Institutet, SE 17177 Stockholm, Sweden; Department of Business Administration, Technology and Social Sciences, Luleå University of Technology, SE 971 87 Luleå, Sweden

**Keywords:** Clinical practice guidelines, Development process, Evidence-based public health, Implementation, Disease prevention, Lifestyle change

## Abstract

**Background:**

Many of the world’s life threatening diseases (e.g. cancer, heart disease, stroke) could be prevented by eliminating life-style habits such as tobacco use, unhealthy diet, physical inactivity and excessive alcohol use. Incorporating evidence-based research on methods to change unhealthy lifestyle habits in clinical practice would be equally valuable. However gaps between guideline development and implementation are well documented, with implications for health care quality, safety and effectiveness. The development phase of guidelines has been shown to be important both for the quality in guideline content and for the success of implementation. There are, however, indications that guidelines related to general disease prevention methods encounter specific barriers compared to guidelines that are diagnosis-specific. In 2011 the Swedish National board for Health and Welfare launched guidelines with a preventive scope. The aim of this study was to investigate how implementation challenges were addressed during the development process of these disease preventive guidelines.

**Methods:**

Seven semi-structured interviews were conducted with members of the guideline development management group. Archival data detailing the guideline development process were also collected and used in the analysis. Qualitative data were analysed using content analysis as the analytical framework.

**Results:**

The study identified several strategies and approaches that were used to address implementation challenges during guideline development. Four themes emerged from the analysis: broad agreements and consensus about scope and purpose; a formalized and structured development procedure; systematic and active involvement of stakeholders; and openness and transparency in the specific guideline development procedure. Additional factors concerning the scope of prevention and the work environment of guideline developers were perceived to influence the possibilities to address implementation issues.

**Conclusions:**

This case study provides examples of how guideline developers perceive and approach the issue of implementation during the development and early launch of prevention guidelines. Models for guideline development could benefit from an initial assessment of how the guideline topic, its target context and stakeholders will affect the upcoming implementation.

## Background

Non-communicable diseases, such as cancer, cardiovascular disease and diabetes are responsible for 60% of all deaths globally, killing roughly 35 million people each year. The WHO estimates that over a third of all cancer cases and up to 80% of heart disease, stroke, and type 2 diabetes cases could be prevented by eliminating known risk factors, such as tobacco use, unhealthy diet, physical inactivity and the harmful use of alcohol [1]. With evidence-based research on how to successfully encourage healthy lifestyle habits, much could be gained, especially if these recommendations could be incorporated into guidelines and clinical care practice. Although the process of collecting, compiling and transferring research evidence into practice in health care settings is challenging, it is imperative to the provision of effective, safe and equitable health care [2,3]. This is particularly true in the fields of health promotion and disease prevention [4].

One way to facilitate the use of evidence in clinical practice is the development of clinical practice guidelines, hereafter referred to simply as ‘guidelines’. Guidelines are defined as “statements that include recommendations intended to optimize patient care that are informed by a systematic review of evidence and an assessment of the benefits and harms of alternative care options” [5]. The principles for developing guidelines have evolved since the 1990s, strongly influenced by values and advances of the evidence-based medicine movement [6]. Guidelines are now being developed at international, national and local levels [7]. The Cochrane Collaboration and the National Institute for Health and Care Excellence (NICE) are examples of organizations that have a long experience of collecting and assessing evidence (which includes both cost of care and health outcomes) to support decisions regarding safe and effective medical and public health interventions [8].

Thus, guidelines are increasingly seen as an effective means to improve health care practices. There are signs of an increased emphasis on both pace and quality guideline development in the past decade, which is exemplified by the emergence of guideline clearing houses (e.g. http://www.guideline.gov/), national programs (Scottish Intercollegiate Guidelines Network) and societies (Guidelines International Network) [9-11].

Scholars have argued that much is to gain if implementation issues are considered early in the guideline development cycle [3]. However, knowledge regarding how guideline developers might address implementation issues is, so far, a relatively unexplored topic in the implementation literature. This study aims to investigate how implementation issues were addressed by the developers of Swedish national disease prevention guidelines during development and early launch phases.

Guideline development can be divided in six phases: prioritizing topics, refining the subject area, assembling development groups, identifying and assessing evidence, translating evidence into clinical practice guidelines and finally reviewing and updating guidelines [12]. Research examining quality of guideline development points to the importance of rigour in the development process, the identification of a target audience, the involvement of health professionals and targeted populations, and the attention to procedures for priority setting and the decision making process [9,13-17].

Several international collaborative initiatives, aiming to increase quality of guideline development, have been undertaken during the last decade (e.g. AGREE Consortium, GRADE working group) [18-21]. The Appraisal of Guidelines Research and Evaluation Protocols I & II (AGREE) was launched in Spring 2000 to improve guideline quality by providing a generic instrument to assess the guideline development process [20,22]. The AGREE domains have also been associated with the degree of guideline implementation [23]. The instrument includes six domains: 1) Scope and purpose; 2) Stakeholder involvement; 3) Rigour of development; 4) Clarity of presentation; 5) Applicability; and 6) Editorial independence. High quality guidelines, as defined by the AGREE protocol, are characterized by their clarity in scope, recommendations and descriptions of target users; high stakeholder involvement during development; rigor of development; and by addressing application issues [23].

Despite considerable investment in developing and disseminating effective guideline recommendations these documents have shown limited influence on health professionals’ behaviour and practice [24-26]. A range of factors appear to impact the use of guidelines in clinical practice [3,24,26,27]. Guideline characteristics [28,29], chosen strategies for implementation and diffusion [2,30,31], and attitudes and motivation of health professionals [24,32] have for example been shown to affect the outcome of the implementation process. The quality of the guideline (e.g. strength of evidence) and the guideline development procedure have also been identified as key factors for increasing guideline credibility and usage [3,26]. Grol and Wensing [33], p.59 suggest that barriers to increasing evidence-based health practices relate to the innovation itself (e.g. advantages in practice, feasibility, credibility, accessibility, attractiveness), the individual professional (e.g. awareness, knowledge, attitude, motivation to change), the patient (e.g. knowledge, skills, health condition, attitude, compliance), the social context (e.g. culture of the network, collaboration, leadership), the organizational context (e.g. organization of care processes, resources, structures) and the economic and political context (e.g. financial arrangements, regulations, policies).

### Guidance on evidence-based, public health interventions

Guidelines which focus on health promotion and/or disease prevention have been in use since the 1980s. Organizations such as the Canadian Task Force on the Periodic Health Examination and the U.S. Preventive Services Task Force, have worked to compile scientific evidence regarding how health services can more effectively engage in disease prevention [34,35]. The National Institute for Health and Care Excellence (NICE) in the UK has pioneered work in this field and has suggested a conceptual framework for addressing social, economic, psychological and biomedical determinants of health and disease among individuals and populations. Their work seeks to understand the casual mechanisms of disease patterns and to utilize a broad approach in public health efforts [36].

Prevention guidelines, in particular, encounter numerous challenges in relation to implementation and use [4]. The early phases of guideline development, such as defining target populations and finding and interpreting the power of evidence, have proven to be particularly difficult, [4]. To be specific, disease prevention guidelines are often built on population-based data, which are typically generic in nature and thus relevant to a range of different disease states. General conclusions about lifestyle recommendations may not be relevant for the specific situation of every individual [4,37,38]. Personal preferences and attitudes towards health promotion among health professionals and patients also affect the use of health-promotion guidelines [32,39].

The systems for decision-making and evidence appraisal in health care are to a large extent similar for both general disease prevention and treatment of specific issues, but the two knowledge areas differ in fundamental matters and the match is not ideal. Evidence-based medicine predominantly uses randomized control trials (RCT) for identifying best practices. Community-level interventions may show significant effects in large populations, but small effects on the individual level. In preventive studies, many RCTs lack adequate power, leading to relatively weak evidence and uncertainty about outcomes [4,40,41].

Differences in the basic principles of evidence-based medicine and evidence-based public health practices have been shown for example in the *span of the evidence.* Public health operates on several levels, which include individual, group, community and societal practices/structures, while medical practice is based on biomedical traditions. The breadth of public health evidence affects guideline development during the search for evidence (e.g. difficulties to find evidence in conventional databases) and the grading of evidence (e.g. combining the principles of assessing the standard evidence in medical science (i.e. RCTs) with the behavioural and social sciences research studies focusing on questions that often do not lend themselves to trial research designs) [40]. Transparent decision models to support the appraisal of public health interventions have been suggested as a means to guide health care decision makers [8].

**Figure 1:**
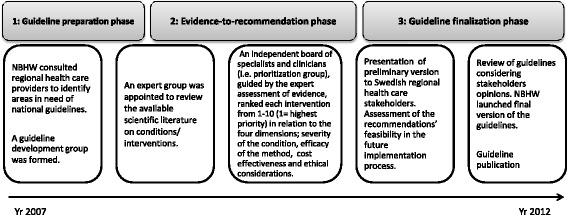
**Guideline development model at the NBHW.**

The National Board for Health and Welfare (NBHW) is the government agency responsible for the development of national guidelines in Sweden. To better promote the implementation of new medical technologies and evidence-based treatments NBHW designed a new model for guideline development in the early 2000s that went beyond evidence compiled by experts. The model included consultations with health care providers, recommendations based on feasibility in the documentation, and prioritization of recommendations based on the compiled, trilateral, evaluation of scientific evidence, health-economic evaluations and ethical considerations (see Figure 1). The formalized procedure for guideline development is described in detail at the NBHW website [42]. The NBHW guideline development policy is intended to create a balance between three dimensions; severity of the condition, efficacy of the method and cost-effectiveness of the method. The NBHW guideline development process deals with problems (e.g. how to increase transparency and inclusiveness in panels and how secure rigour and quality in its methodology) identified also by other guideline developers. The model adopts similar types of solutions as those utilized by larger guideline developers in other countries [36,43-46]. For example, NICE has addressed issues on systematic use of research evidence and assessing cost-effectiveness and stakeholder involvement. These are fundamental guideline development features that also are included in the NBHW model [42,46]. The NBHW guideline development model is frequently updated to improve and enhance guideline quality in guideline production [42].

The NBHW guideline development process follows a transparent methodology (documented in methods manuals) that states how evidence should be collected, assessed and transformed into recommendations. The priority of an intervention is set by assessing the gravity of the related, unhealthy lifestyle practice (e.g. smoking) and expected benefits and costs of the intervention (e.g. advanced counselling) based on scientific evidence and evaluations of cost-effectiveness [47].

The NBHW model was based on the premise that Swedish health care, by law, is considered to be a responsibility of all the county councils and municipalities in the country. The Swedish county councils also have the primary responsibility for guideline implementation at the regional level and for deciding the level of resources which will be made available in clinical practice. Target users of guidelines were primarily health care decision-makers, but also health professionals and patients treated for other reasons.

Since 2003, NBHW has developed 15 national guidelines, mainly focusing on treatment of medical conditions. Several of these guidelines indicate that changes in unhealthy habits are important. However, they do not discuss which methods are best suited for this purpose. In 2008, NBHW began the process of developing guidelines for disease prevention – the National Guidelines for Methods of Preventing Disease in Clinical Practice, hereafter called Disease Prevention Guidelines. These guidelines focused on four lifestyle habits: tobacco use, hazardous use of alcohol, insufficient physical activity and unhealthy eating habits.

The aim of this study was to investigate how implementation challenges were addressed during the process of developing guidelines for disease prevention. These Disease Prevention Guidelines offered the opportunity to study both general and prevention specific implementation challenges, as well as strategies used by government agency guideline developers.

## Methods

This case study is based on interviews conducted with guideline developers involved in the creation of the NBHWs Disease Prevention Guidelines, as well as on archival data. Data were collected between Oct. 2009 and Sept. 2012.

### Data collection

Seven semi-structured interviews were conducted with the guideline development management group and the head of the guideline development unit at the NBHW. Interviews focused on the Swedish model for developing national guidelines and explored how implementation challenges were perceived and handled during the guideline development process. An interview guide attempted to capture guideline developers’ experiences and their thoughts regarding implementation challenges. The guide also included questions for exploring strategies for addressing challenges during the guideline development process. Examples of interview questions include: Could you describe the process for developing guidelines on disease prevention, from when you started, up to the present moment? How did the NBHW’s general guideline development model apply in this specific case? What do you perceive the major challenges will be for ensuring that guidelines reach targeted users? What role should NBHW play in regard to implementation of the guidelines? Interviews were conducted with all members of the guideline development management group and the head of the department of guideline development at the NBHW. Interviews lasted from 55 to 95 minutes. They were recorded and transcribed verbatim and respondents were asked to review the transcripts to identify any necessary corrections.

Additionally, archival data were collected from the NBHW website (e.g. the final Disease Prevention Guidelines document and information sheets), from official documents (e.g. laws, government bill) and through personal contacts with the guideline developers at the NBHW (e.g. formal steering documents concerning the general guideline development process and the NBHW).

### Data analysis

Qualitative content analysis [48] was used to analyse qualitative data. The analysis procedure was performed in five steps. First, transcribed data from interviews and texts in archival data were read through several times to get a sense of the overall content. Secondly, content that referred to implementation challenges and strategies (e.g. barriers and facilitators of implementation, activities and strategies aimed to promote implementation) was identified for further analysis. In a third step, text units with coherent statements (i.e. sentences, sections of text with coherent content) were coded with a condensed label intended to capture the essence of meaning. Codes with a common meaning were then grouped in categories. In a fourth step the categories with a common meaning were merged into themes. Finally, the themes were structured in a theoretical framework inspired by the domains in the AGREE II instrument [22]. The domains reflect significant aspects of Guideline development and have implication for their prospect to be implemented in practice. The themes that were identified in the analysis process (step II-IV) were organized into one of the six AGREE domains in a table. The themes that did not fit the AGREE framework, are described separately in the results section. Data analysis was performed by two researchers (LRS, TK). When opinions differed, discussion with a third researcher (MN) was performed to reach consensus and if consensus could not be reached the decision was based on the views of the majority.

### Ethical considerations

Participation was based on informed consent and was voluntary. The study was approved by the regional Ethics Committee at Umeå University, Umeå, Sweden [Dnr 2011-64-31 M].

## Results

Our analysis of the development of the Disease Prevention Guidelines shows that implementation challenges were identified and addressed by guideline developers. Several of these activities and approaches i could be condensed into four themes: 1) Broad agreements and consensus about scope and purpose; 2) Systematic and active involvement of stakeholders; 3) Formalized and structured development procedure; and 4) Openness and transparency in the specific guideline development procedure. Two additional factors were perceived as influencing developers’ choices for addressing implementation issues; the preventive guideline scope and guideline developers workload. Table 1 presents an overview of the identified strategies organized within the AGREE domains. Statements from interview respondents and from the final version of the Disease Prevention Guidelines are used to exemplify and clarify themes and categories.

**Table 1:** **Strategies to address implementation challenges during development of the Disease Prevention Guidelines**

**Framework**	**Theme**	**Category**	**Sub-category**
Guideline scope and purpose	Broad agreements and consensus about scope and purpose	Target audience involved in framing the guideline scope and purpose	Hearings with health-promoting organizations and politicians, professional groups
			Political proposal preceded guidelines with health-promoting scope
	Systematic and active involvement of stakeholders		Health-promoting hospitals networks used for piloting scope and identifying target population of Disease Prevention Guidelines
	Formalized and structured development procedure	Using systematic methodology for identifying and defining the Disease Prevention Guidelines concepts and health questions	Formal criteria for initiating CPG-process
			Preparatory development process defining concepts and scope
			Systematic methodology in defining guideline health questions and key concepts
Stakeholder involvement	Systematic and active involvement of stakeholders	Guideline development group included a wide scope of relevant professional groups and fields of knowledge	Experts representing: the CPG target area/s; methodology; communication; ethics; health economics
			Health professionals representing: the CPG target area/s; different parts of the country; different patient groups; and different parts of the health care system
		Involvement of stakeholder throughout the guideline development process	Meetings and collaboration with health-promoting organizations, politicians, and professional groups in the early phases of development
			Regional conferences with health providers in the county councils
Rigour of development	Formalized and structured development procedure	Formalized CPG development procedure	Formalized procedure for defining purpose, scope and concepts
			Formalized procedure for searching and assessing evidence
			Formalized consensus procedure when there is a lack of evidence
			Formalized procedures are suggested for monitoring, evaluation and follow- up of Disease Prevention Guidelines
	Openness and transparency in Disease Prevention Guidelines development procedure	Transparency in methodology	Guideline development procedures explicit and overt
			Recommendations and the supporting evidence are clearly connected
Clarity of presentation	Openness and transparency in Disease Prevention Guidelines development procedure	Clear presentation of guideline development model	Description of the general Guideline development model is presented
			Description of the Disease Prevention Guidelines development model and organization is presented
			Methodology for collecting evidence is presented
		Clear presentation of the Disease Prevention Guidelines recommendations	Experts in communication involved in the formulation of Disease Prevention Guidelines
			Links between recommendations and evidence are clear
Applicability	Systematic and active involvement of stakeholders	Target users tested the hypothetical use of the Disease Prevention Guidelines recommendations	Health professionals hypothetical tested the use of the Disease Prevention Guidelines in clinical setting
			Health care providers hypothetical tested the use of the Disease Prevention Guidelines in a health care management setting
	Openness and transparency in Disease Prevention Guidelines development procedure	Barriers/facilitators for the Disease Prevention Guidelines application were presented	Facilitating factors for the implementation of the Disease Prevention Guidelines were sought by target users
		Monitoring, evaluation and update of Disease Prevention Guidelines	Barriers for the implementation of the Disease Prevention Guidelines were sought by target users
			Formalized procedures for monitoring, evaluation and follow- up of Disease Prevention Guidelines are suggested
Editorial independence	Openness and transparency in Disease Prevention Guidelines development procedure	Autonomy of Guideline developers	The NBHW autonomous government agency
			Formalized procedures for seeking and recording possible competing interests of all members of development group

### Broad consensus about the guidelines scope and purpose

Archival data state that the need for disease preventive guidelines was initially raised as a political initiative, based on national public health policy. This policy was launched by the Swedish Parliament in 2002 and specifically targeted the role and responsibility of the health service providers to improve their performance in health promotion [49]. In parallel, the NBHW identified large regional differences in Swedish health care provider disease prevention practices. To meet these challenges, NBHW decided to develop national guidelines that would focus on disease prevention.

Respondents described how hearings and spoken agreements with a broad range of interest groups (i.e. health care providers, politicians) were used to identify and settle on a feasible focus and objectives for the Disease Prevention Guidelines. The developers considered these collaborations and agreements to be a significant component for implementation success, as they prompted a readiness, both among guideline developers and among target users, on what the guidelines may come to imply.

“The goal was to reach consensus over the country about which areas these guidelines should address as early as possible in the process. To direct health care in a top-down manner on how they should work. But guidelines are after all a soft law, there is no support in the (formal) law. The NBHW cannot give penalties to health providers who do not adhere to the guidelines. If we do not collaborate with the users – in a broad sense – from the very beginning, clinical guidelines will stay on the bookshelves. If we fail to narrow them down and to have a realistic scope the main objective also fails, to reduce disparities across the country and the differences in the various groups of the population access to care.” (Guideline developer, Disease Prevention Guidelines).

### Systematic and active involvement of stakeholders

**Figure 2:**
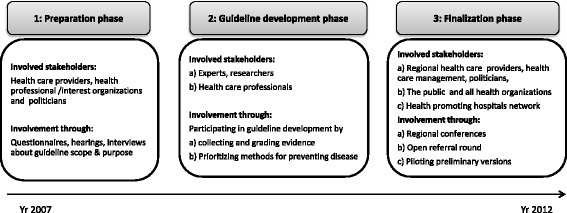
**Involved stakeholders in different phases of the guideline development.**

Steering documents and respondents described how different actors were involved in the process during different stages of the development process (Figure 2). According to the NBHW guideline development model, caregivers should be regularly involved during the process. Respondents described how this was particularly important in the case of the Disease Prevention Guidelines as it, unlike diagnosis-related guidelines, has no easily identified target population.

The Disease Prevention Guideline development involved 70 individuals organized in six operative groups: the guideline development management group (6), a prioritization group (25) and four expert groups (39). The expert groups were staffed based on their expertise in prevention and one or more of the four life-style areas. Expertise in the fields of scientific methodology, health economics, ethics and communication/linguistics were also made available to the guideline development group.

Health professionals, experienced in disease prevention in clinical settings, were involved in the process of prioritizing. The task of the prioritizing group was to rank recommended practices from 1 (highly recommended) to 10 (not recommended) based on the severity of the condition, efficacy of the method, and cost-effectiveness. With the goal of involving a variety of stakeholders, the developers tried to obtain wide representation in the prioritization group, equal gender representation, and accordingly engaged professionals from different parts of the country, professional groups and knowledge areas. A majority were also trained researchers. The meetings with the prioritization group were organized to facilitate active participation with a decision process described as an adopted Delphi-method.

The purpose of involving many people in the development process was associated with the implementation challenge. The network activities aimed to create broad agreements about the advantages and demarcations of disease preventing guidelines, as early as possible in the process. Secondly collaborations aimed to raise awareness about the upcoming guidelines and to engage influential actors in the early stages of guideline development. Finally, by including representatives of many different stakeholder groups the developers hoped to predict and plan for a wider range of implementation barriers.

Throughout the development process influential organizations and networks (e.g. NGOs, public authorities and a national network of health-promoting hospitals) were involved through hearings, interviews, and piloting trials.

“By collaborating with the health professional groups and the national network of health-promoting hospitals and incorporating them in our development process, in defining the main purpose and what lifestyle areas we should include and which we had to exclude, we aimed to plant a seed of change that would have the opportunity to develop as the guidelines developed. The initial efforts to identify the relevant health questions are crucial for implementation. And that starts on day one of the guideline development, with hearings and broad discussions. We thought ‘implementation’ from the very beginning.” (Guideline developer, Disease Prevention Guidelines).

Respondents also described difficulties with involving the target population in the guideline development process. Traditionally, NBHW utilized patient/consumer organizations to recruit target populations (e.g. patients, public). However, in the case of the development of Disease Prevention Guidelines, recruitment was difficult due to the focus on prevention. Respondents indicated that the target populations’ perspective was, to some extent, advocated by the health professionals serving in the prioritization group.

During the final stages of guideline development, potential target users were involved in regional seminars and referral rounds where stakeholder views and preferences were sought. These views and preferences led to changes in the final version of the guidelines. Respondents described how stakeholder groups (e.g. health providers and representatives from the health promoting hospitals network) raised different considerations regarding the future use of the guidelines. For example, stakeholders asked which health professional groups (e.g. physiotherapists, nurses, GPs, psychologists) would be responsible for the interventions recommended in the guidelines. Developers chose not to provide detailed answers about application of the guidelines, as they felt this was the most appropriate response in light of health providers’ independence from national governance. Respondents also argued that this standpoint stimulated local engagement and responsibility for the implementation of the guidelines.

### Formalized and structured development procedure

The NBHW guideline development model outlines a formalized procedure for preparing and completing the development of guidelines. Respondent comments underscored the need for a proscribed process and described how the definition of key guideline concepts required a systematic, preparatory procedure, where the basic concepts and terms were thoroughly scrutinized and defined. Respondents attributed the clarity of scope, purpose and concepts as important for encouraging target user’s motivation, understanding and future accuracy in guideline usage.

“The concepts of health promotion or disease prevention were not very distinctly defined among us when the development of the Disease Prevention Guidelines was initiated. And if we were not clear about these basic concepts in the very beginning, we acknowledged the obvious risk that the guidelines would not be used as we or the literature we based the recommendations on intended. So we put a lot of energy and resources in defining the concepts and scope of the guidelines.” (Guideline developer, Disease Prevention Guidelines).

Procedures for searching, assessing evidence and formulating recommendations were described in the model and in the final version of the Disease Prevention Guidelines and published on the NBHW website. A plan for evaluating and updating guidelines was provided with the final version of the guidelines. The guideline developers identified the use of systematic, transparent and scientifically sound methods as an essential factor for implementation, affecting future guideline users’ perception of the reliability and trustworthiness of the Disease Prevention Guidelines. Respondents stated that for some conditions/interventions there was a significant lack of evidence for appropriate grading approaches (according to GRADE). This problem was addressed through a formalized consensus procedure where existing evidence was complemented with proven, clinical experience.

“It is easier to develop guidelines with a medical scope since they often include a higher amount of studies that fit our questions as developers, compared to guidelines with a preventive scope. It is more difficult when we do not have a good scientific basis and we have no health economic data. It is a flaw. Then it may be based on estimates, which is not optimal. We have handled the shortcomings of scientific data through developing a consensus process. We had a need to formalize and systematize proven experience in any sense.” (Guideline developer, Disease Prevention Guidelines).

Respondents also described how the structured and rigorous guideline development process was demanding for the members of the guideline development groups. Respondents expressed that that it was a challenge to educate the participating experts and health professionals on the development model and follow it through.

### Transparency in the disease prevention guideline development process

Several of the respondents described efforts to provide a rich description of Disease Prevention Guidelines development methods, as well as providing evidence and the health economic evaluations to clarify the basis for recommendations. The recommendations were linked to references and the background material and the final guideline document was published on the NBHW website. Respondents felt that these considerations were important for increasing transparency and reliability in the development process. Respondents also critiqued their own performance in the development process concerning ambiguous instructions to involved experts and health professionals. Respondents felt, that this issue provide some explanation for the delay in guideline completion. The process took five years and was delayed approximately ten months in relation to the original completion deadline.

Communications and linguistics experts edited the final document to strengthen clarity and reduce ambiguities. Recommendations were described in summary form and included brief advice, counselling or qualified counselling as methods for changing lifestyle. The recommendations included a brief description of counselling methods, with more detailed descriptions given in a supplement. The guideline developers stated that it was very difficult to keep a balance between being clear and specific and being general enough to preserve the independence of the Swedish regional health care providers.

The respondents underscored the value of the regional conferences as a means for addressing implementation challenges in the last phase of the guideline development process. In these conferences, regional health care providers and guideline developers met and openly assessed the implications of the Disease Prevention Guidelines. Some of the respondents stated that the regional conferences increased the health care providers’ awareness and responsiveness to the final version of the guidelines. Resource implications and potential implementation barriers were presented by health care providers during the final phases of the development process. By asking health care managers to assess consequences and resource implications of guideline implementation (e.g. educational, technical resources) they hoped to influence the target users to use of the Disease Prevention Guidelines at local, regional and national levels. Thanks to these regional considerations, a chapter detailing financial and organizational consequences of guideline implementation was presented, discussed and included in the final version of the Disease Prevention Guidelines.

“The NBHW concluded that the guidelines required (organizational) changes in terms of human resources, steering documents, organization and reallocation of resources, education and skills and interaction with stakeholders within and outside the health sector.” (text extracted from the Disease Prevention Guidelines).

Respondents also discussed how guideline developers (including experts and health professionals) were asked to state their competing interests. Information about all members of the guideline development group was documented in the initial phase of the Disease Prevention Guidelines process and one member was excluded from the process based on the possibility of editorial dependence. The respondents felt these efforts to ensure editorial independence, increased guideline credibility, which in turn would enhance guideline implementation.

### Additional perspectives on implementation during guideline development

Respondents described the process of disease prevention guideline development as challenging and explorative and a pioneering journey. The focus on prevention, challenged various components of the established model for guideline development at NBHW, being that is was a new type of guideline (preventive) that covered a large field and several topics (four, lifestyle habits). The generic characteristics of these prevention guidelines also created challenges relating to the multidisciplinary audience of guideline users and target population. Respondents described the process of defining the intended users and target population (i.e. health professionals and individuals with hazardous lifestyle/s) as long and complex. Specific attention was given to this issue and criteria for identifying the target population were developed and piloted through the health-promoting hospitals network in Sweden.

The long and complex process of guideline development was largely undertaken by the 3–4 people in the guideline development management group. Towards the end of the process, the workload increased and respondents described this last phase of development as intense and very demanding. Some respondents concluded that the strained work conditions led to a decreased focus on implementation and guideline applicability, thereby challenging developers’ ability to plan ahead in the process.

## Discussion

Several key issues influencing the success or failure of guideline implementation are settled already during the guideline development process. It is therefore urgent to address these issues when planning for guideline development. Several basic features (e.g. the purpose of guidelines, resource implications, clarity of recommendations) have been shown to play an important role in guideline usage [3,22], which suggests that the development phase offers great opportunities to improve the implementation of guidelines. How this should actually be carried out in practice, is a topic that has received less scrutiny.

This case study provides an in-depth look at the possibilities to address implementation challenges during development of prevention guidelines. Several activities and approaches for enhancing the achievement of guideline intentions were identified. Four areas for achieving guideline development success included: broad agreements and consensus about scope and purpose of the guidelines, a formalized and structured development procedure, systematic and active involvement of stakeholders, and openness and transparency in the guideline development process. In addition, the early involvement of the target audience and the involvement of a broad group of stakeholders made hypothetical trials and discussions of guideline implementation possible. This was one aspect of the development process that seemed to be especially important for prevention guidelines. To increase trustworthiness and the quality of the guidelines, rigorous and transparent methods were used for sourcing, prioritizing and incorporating information on health economics and ethics.

The NBHW’s main task is to develop guidelines and recommendations in a credible way and, through collaborations, transparency and support, to stimulate implementation. However, the NBHW is restricted in its influence, due to a limited mandate as a government agency among rather autonomous county councils and municipalities in the Swedish health care system.

To be used, it has been argued that clinical guidelines need to be established and integrated as a part of other health care quality improvement processes [50]. This requires cooperation between guideline developers and health care’s stakeholders [51] In this case the planning and development of guidelines involved both a national authority and the regional and local care providers. Such integration is possible if consensus on focus and purpose can be accomplished. Having a dialogue and making agreements with stakeholders at different levels in the health services system – from politicians to health care providers and health professionals, is also a valuable practice in the development process.

Further, this strategy of broad agreement may have contributed to raised awareness and motivation among guideline target users (e.g. health care management) in a way that boosts health care organizational readiness to alter their course in the direction of the Disease Prevention Guidelines. This strategy is supported by similar research. For example, Cabana et al. [24] identified lack of familiarity, awareness and agreement with guidelines as significant barriers to guideline adherence among physicians. Nevertheless the involvement of a large number of stakeholders, the balancing of integrity with the NBHW guideline model, and the flexibility introduced by the stakeholders’ involvement, all came at a cost. The process was described as complex, long and demanding by the guideline development management group. This reflects a need to regularly address process issues, such as the time and resources required in different development phases and type of complexity connected to various guideline areas.

The NBHW used its standard guideline development model with small adjustments to fit the prevention focus. The unusually complex development process that respondents found frustrating might reflect difficulties that come with matching public health interventions to the prevailing medical evidence system [8,40,41].

The involvement of patients or other target populations in guideline development is supported by WHO, the Guidelines International Network and AGREE [15,22,52]. However, recent studies have shown that methods for increasing target user engagement still need to be improved [53]. For diagnosis-related guidelines, the Swedish model systematically involves patient organizations in the development process. This was not applicable with regard to Disease Prevention Guidelines as the “Patient Organization of the Not Yet Diseased” does not exist. Due to its generic focus, the lack of clear stakeholders and target users may have hindered involvement of these groups during development. Instead, in the Swedish context, elected representatives, primarily politically designated providers, were assumed to take on the role of representing the public.

Our results are based on a single case study of prevention guideline development in a specific national context. There might be other aspects that need to be considered in other contexts and other ways of achieving a more conscious focus on implementation issues and preparations for implementation.

## Conclusions

This case study provides examples of how guideline developers perceive and approach the issue of implementation at an early stage in the life-cycle of prevention guideline development. The study also identifies characteristics of the process of developing guidelines with a preventive or public health focus. These include the complexity of addressing implementation issues in this area and the need for a decision model when addressing research not based on RCTs. The prevention focus influenced how implementation challenges were perceived and addressed. We conclude that guideline developers could benefit from including an assessment of what the specific focus areas (e.g. preventive, treatment) of guidelines may imply for their future use and how these areas impact the support needed for guideline implementation. Another question that should be explored in future research relates to how scientific evidence from the field of public health and preventive medicine should be operationalized and assessed in the guideline development process.

It seems obvious that societies would benefit from utilizing available knowledge for addressing major threats to global health and innovative ways for improving life-style practices of large populations. But the path from evidence to changed health practice is an uneven one. Prevention guidelines might aid this process, but clinical guidelines will reach their potential only if they are known, adopted and used by their target groups. By illustrating how specific implementation challenges were perceived and addressed throughout different phases of guideline development, this study can provide some ideas for how to improve guideline development and support the implementation of preventive actions in health systems and health care practices.

## Abbreviations

AGREE: Appraisal of Guidelines for Research and Evaluation
GP: General Practitioner
GRADE: The Grading of Recommendations Assessment, Development and Evaluation
NBHW: National Board of Health and Welfare in Sweden
NICE: The National Institute for Health and Care Excellence in the UK
RCT: Randomized Controlled Trial
WHO: World Health Organization
